# Edge-Aided Reliable Data Transmission for Heterogeneous Edge-IoT Sensor Networks

**DOI:** 10.3390/s19092078

**Published:** 2019-05-05

**Authors:** Zhirong Xu, Ming Cai, Xiaoyan Li, Tianlei Hu, Qianshu Song

**Affiliations:** 1 College of Computer Science, Zhejiang University, #38, Zheda Road, Hangzhou 310024, China; 11021034@zju.edu.cn (Z.X.); htl@zju.edu.cn (T.H.); 2 Netease (Hangzhou) Network Co., Ltd., Hangzhou 310052, China; lixiaoyan@corp.netease.com; 3 Administrative Office, Zhejiang Association for Science and Technology, Hangzhou 310006, China; negosong@163.com

**Keywords:** sensor networks, edge-aided computing, data transmission, reliability

## Abstract

Wireless sensor networks have been attracting research attention for the past decade and will continue to be a hot topic due to the emerging trend of Internet-of-Things (IoT). Edge computing for IoT (Edge-IoT) is a promising framework that can help low-powered sensor networks to conduct complex computational tasks. Different from the existing works that focus on cooperative task execution for edge and sensor networks, in this paper, we investigate the problem of reliable data transmission in edge-aided sensor networks. Firstly, we discuss how edge servers can help to improve the data transmission of sensor networks. Secondly, we propose a forwarding scheme for edge nodes to forward packets according to coverage and corresponding interference. Thirdly, we propose an edge-based error recovery approach. By employing edge servers for data transmission and error recovery, the efficiency and reliability of data transmissions can be largely improved.

## 1. Introduction

The recent advances of low-power wireless communications and computation have led to the development of low power sensor networks (WSNs) for the Internet of Things (IoT). As various mobile applications have emerged and attracted much attention from both research and industry, WSNs are also attracting more and more research efforts [1,2]. A typical WSN consists of a (large) number of resource-constrained sensor nodes equipped with low-power sensors, CPUs and radios. WSN-based applications cover large-area monitoring [3,4], smart grid [1], health care [5,6], smart homes [7], green buildings [8,9], etc. Different from the existing works on sensor networks, the IoT oriented WSNs are often required to undertake more computational and communicational tasks [1]. For example, in smart city scenarios, sensor networks can be used for counting crowd or detecting people’s identities [10,11].

To empower sensor networks to fulfil the complex and massive tasks, edge computing [12] is a promising direction. Edge computing is a new paradigm which adds additional edge computing servers to low-powered devices and networks [13]. The computational tasks from the underlying users (devices/sensors/mobile users) are firstly offloaded to the edge computing servers [14], and the edge computing servers become responsible for the execution of those tasks. The tasks can be transferred to the edge computing servers via LTE networks, WiFi networks, or low-powered communications technologies [15]. The tasks received by the edge computing servers are handled by task assignment schemes [16] and placed at certain edge computing nodes. The process differs according to the different edge architecture, such as user-edge framework [13], user-edge-edge framework [17] and edge-cloud coordination [18]. In cases where there are not enough edge resources for the tasks in edge-cloud coordination, part of the tasks are uploaded to the remote cloud servers [18].

Edge-aided sensor networks [13] combine edge computing and sensor networks to increase the computational capacity of sensor networks. The tasks from sensor networks can be fully or partly uploaded to the edge servers, and then the edge servers return the computational results to the sensor nodes. Some existing works have been proposed to optimize the cooperative task execution in edge-aided sensor networks. Wang et al. propose to assign the offloaded tasks to specific edge servers [16]. Zhao et al. propose to deploy edge servers to a random IoT network, considering both communication and computation requirements [19]. Chen et al. manages the task offloading for ultra-dense wireless networks [17].

While these works focus on the task execution and resource optimization, we argue that edge computing servers can also be a very powerful tool to improve the data transmissions in sensor networks in terms of both efficiency and reliability. There are a number of existing works on reliable data transmissions in sensor networks. For example, Seda [20] retransmits partial packet payloads to receivers when packet corruptions happen. TinyRS [21] employs the RS code to establish effective error correction for sensor networks. ZiXOR [22] uses XOR codes to adaptively recover transmission errors in a lightweight manner.

Compared to the existing works, edge-aided sensor networks have more potential to further increase the energy efficiency of data transmissions. Specifically, edge servers often have much more powerful radios and have an unlimited power supply (while sensor nodes are powered by batteries [23]). Therefore, if the transmissions are migrated from sensor nodes to the edge servers, much energy and delay overheads can be saved and the lifetime of sensor networks can be increased.

To exploit the chance of edge computing servers, there are two challenges. First, the coverage of edge servers is much larger than that of a sensor node. As a result, when an edge server transmits a packet to a certain number of sensor nodes, it causes interference to other sensor nodes which do not need those transmissions. Whether it is beneficial or harmful for the overall performance of data transmissions is the key to deciding whether we should employ edge servers or not. Second, for the poor links in sensor networks, error recovery from sensor nodes may cost a large number of retransmissions. At the same time, if we use edge servers to recover those packet errors, much overhead is saved. A key challenge for retransmissions of edge servers is that edge servers are not aware of the real-time status of packet receptions/losses. The acknowledgments from sensor nodes cannot be delivered to the edge servers due to the limited communication range of sensor nodes.

To overcome the above challenges, we propose an edge-aided reliable data transmission scheme for heterogeneous sensor networks. First, we devise a fast estimation scheme to help edge servers to evaluate how many sensor nodes are requiring the current packet. On the basis of the estimation, we further estimate the potential benefits of using edge servers for data transmission. If the improved packet reception rate (PRR) for target sensor nodes is larger than the degraded PRR for other sensor nodes, the edge server starts forwarding the data. Otherwise, the edge server drops those packets until the above condition is met. Second, we propose a PRR-based reception estimation scheme to find out how many sensor nodes have missed packet transmissions multiple times with poor links. When the number of missing packets exceeds a given threshold, the edge servers start packet retransmissions to avoid endless packet retransmissions on poor wireless links. On the basis of the above two novel schemes, we design a framework of edge-aided reliable data transmission and implement it in simulations. The evaluation results show that with the help of edge computing servers, the efficiency of data transmissions in sensor networks can be largely improved.

The major contributions include:

1. The identification of the opportunity of exploiting edge servers to increase the reliability of data transmissions in sensor networks.
2. The proposal of an adaptive approach to employ edge servers for data transmissions. The edge servers are involved in data transmissions only when it is beneficial and the overall reliability of data transmissions in the sensor network can be improved.
3. The proposal of an edge-aided retransmission scheme to further increase the reliability of *poor* links in sensor networks, with which the number of retransmissions can be significantly reduced.

The remainder of this paper is organized as follows. Section 2 presents the related works of data transmissions in sensor networks and recent advances in edge computing. Section 3 presents the main design of the edge-aided data transmissions in heterogeneous sensor networks. Section 4 evaluates edge-aided data transmission with simulation experiments. Section 5 concludes this work and discusses future directions.

## 2. Related Works

In this section, we introduce the related works on data transmissions in sensor networks. Additonally, we discuss the recent advances in edge computing for sensor networks. After that, we compare our work and the existing works to find out the most suitable scenarios for our work.

### 2.1. Reliable Data Transmissions in Sensor Networks

Reliable data transmission is a well-studied problem in sensor networks. To deal with possible packet losses, there are basically two solutions: Reliable routing and error recovery. In reliable routing, each node selects the most reliable links for data transmission such that potential interference can be avoided [4]. Gnawali et al. [24] propose a typical routing protocol in ad-hoc networks which selects the node with smallest expected number of transmissions (ETX) as the next-hop forwarder for each hop. ECD [25] additionally considers different link features such as link quality and link correlation to improve the transmission efficiency and reliability. CD [26] is an efficient dissemination scheme (wireless multicast) to improve the reliability and reduce propagation delay of data transmissions. CoCo and CoCo+ [27,28] establish a fixed network structure for efficient multicast in sensor networks, where link features and transmission progress are both considered. In error recovery, some works use retransmissions to deal with packet losses and some other works employ coding schemes such as network coding and forward error correction codes for reliable data transmissions. Seda [20] is a partial retransmission scheme to save the transmission cost in sensor networks. ULTRA [29] is an out-of-order data transmission scheme for bulk data transfer, where all packets can be delivered to the destination nodes without following strict sequences. TinyRS [21] employs FEC codes to improve the packet reception rate for wireless transmissions. ZiXOR [22] is a lightweight coding scheme to reduce the coding delay and improve the reliability of data transmissions. These works do not rely on extra resources. However, in edge computing for IoT (edge-IoT) networks, edge servers are deployed throughout the network and can be used as potential senders to forward and retransmit data packets. As a result, in such a new network paradigm, the existing works on data retransmissions and error correction schemes cannot be directly employed.

### 2.2. Edge Computing for Sensor Networks

Edge computing is a novel computing paradigm that provides a powerful solution to the resource-constrained sensor networks [13]. Edge servers are deployed in the sensor network such that the sensor nodes can offload the computational tasks to the edge servers and save much energy and computational delay. In this way, the sensor network can undertake much more complex computation tasks and much more tasks than traditional sensor networks.

The existing works on edge computing for sensor networks mainly focus on the task offloading [17], resource allocation [16], and edge deployment [19]. In [17], the novel architecture of using edge servers to empower the low-power IoT networks is proposed. In [16], an adaptive scheme to offload the tasks from embedded devices to edge servers is proposed. The tasks are generalized as directed acyclic graphs (DAGs) and the dependency among different sub-tasks are considered. In [19], a task assignment scheme to allocate the task to the most appropriate edge servers considering the mobility of the underlying sensor nodes is proposed.

### 2.3. Summary

The basic idea of the existing works is to employ the computing resources of the edge servers to empower sensor networks and help sensor networks to fulfill more complex tasks. Different from the existing works, our basic idea is to seize the opportunity to exploit the additional communication abilities of edge servers to improve the reliability and efficiency of data transmissions in sensor networks. As edge servers are not constrained by battery capacity, it can use much larger radio power to transmit data packets, which covers more nodes than traditional low power radios on sensor nodes.

Specifically, we exploit both forwarding and retransmissions to improve the reliability and energy efficiency of data transmission in sensor networks. The packet forwarding can save a number of transmissions of sensor nodes. In addition, for those poor links with very low packet reception rates, the retransmissions from edge servers can also reduce delay overheads and transmission to a great degree.

## 3. System Design

In this section, we propose the main design of the edge-aided data transmissions in sensor networks.

### 3.1. System Model

**Figure 1:**
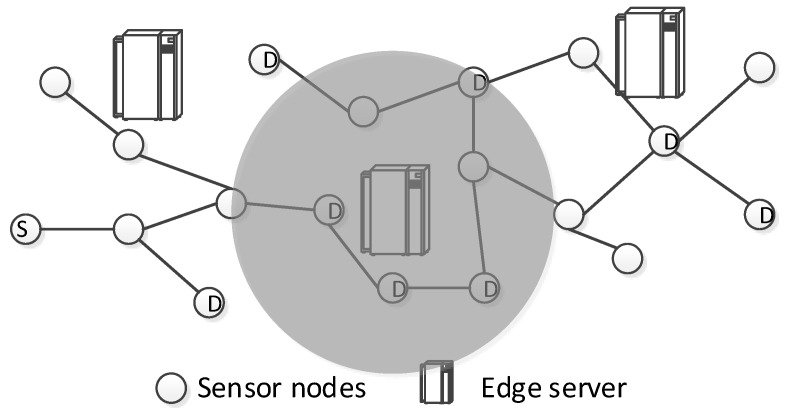
The system model of edge-aided sensor networks.

We follow the existing works on the combination of edge computing and sensor networks. The system model is shown in Figure 1. In the heterogeneous sensor networks, various different kinds of applications and computational tasks co-exist. In the area of the sensor networks, a number of edge computing servers are deployed to provide computing services for the underlying sensor nodes. The communication range of the edge servers is much larger than the communication range of sensor nodes as the edge servers have no battery limit. The radios on edge servers can be set to the highest power level while the sensor nodes need to reduce the power level to save energy.

**Task model.** We assume a one-to-many model of data transmissions in sensor networks [30]. In sensor networks, maintenance is one of the most important problems, where information needs to be distributed from one node to a number of different other nodes. We focus on such scenarios as shown in Figure 1. Node *s* intends to transmit controlling packets to all destination nodes, which are marked as *d*.

**Data transmission model.** In traditional sensor networks, node *s* will deliver the packets in a multi-hop manner and eventually cover all the destination nodes. In the edge-aided sensor networks, when the packets propagate to the area of edge servers, we may employ the edge servers for data transmissions such that many hops can be saved. For example, in the figure, if we use the edge server (in the middle) to forward the packet, four destination nodes are covered immediately and at least four relay transmissions are saved for each packet.

**Retransmission model.** We use the Acknowledgment-based (ACK-based) retransmission mechanism. When a sensor node receives a packet, it will return a sequenced ACK to its sender. The sender will retransmit the packet if the ACK is timed out. For poor wireless links, the edge servers will transmit packets to the target sensors. The key challenge is that the communication range of sensor networks is highly limited compared to the communication range of edge servers. The edge servers may not be aware of the poor links with multiple retransmissions. We detail our solution to this problem in Section 3.4.

### 3.2. Framework

**Figure 2:**
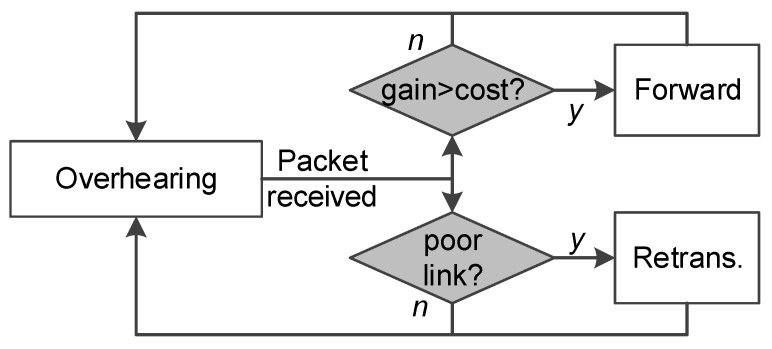
The framework: The edge servers can empower data transmission in sensor networks via *Forwarding* and *retransmissions*.

We propose an edge-aided data transmission scheme for sensor networks. Figure 2 shows the framework. We employ the edge servers to improve the transmission efficiency in two ways: data forwarding and data retransmissions.

**Data forwarding.** When an edge server overhears a packet transmission nearby, it first finds out the destination nodes of the current packet. Then, the edge server estimates the potential benefits if it transmits this packet. On one hand, the destination nodes have more chance to receive the target packets; On the other hand, the non-destination nodes in the edge’s communication range experience severe interference. The estimation of the potential benefits must consider both the improved gains and possible costs on all nodes in its communication range. The details of the forwarding mechanism are discussed in Section 3.3.

**Retransmissions.** The edge servers overhear nearby transmissions and identify those repeating retransmissions. When some packets are retransmitted multiple times, it is highly possible that the link is temporarily down or is of poor quality. In such cases, the edge nodes may adaptively help retransmit the packets to the target destination node(s). The details of the retransmission mechanism are discussed in Section 3.4.

With the forwarding and retransmission schemes, the efficiency and reliability of data transmissions in sensor networks are significantly improved.

### 3.3. Edge-Aided Data Forwarding

In this subsection, we detail the forwarding of edge servers. When a packet is overheard by the edge server, it estimates the potential gain of forwarding the current packet. We define the gain as the expected number of saved transmissions over the number of interfered nodes. The gain, denoted as *g*, is then calculated as

(1) $$g=n_{s}-n_{i}$$

where $n_{s}$ denotes the number of saved transmissions, and $n_{i}$ denotes the number of interfered nodes. $n_{s}$ is calculated as

(2) $$n_{s}=\sum_{k=1}^{|N_{c}|}q_{k}$$

where $N_{c}$ denotes the set of destination nodes covered by the edge server, and $q_{k}$ denotes the link quality from the edge server to node *k*. For the measurement of $q_{k}$, we can follow the existing works to reduce the measurement delay such as in [31].

Then, we calculate the number of interfered nodes, $n_{i}$, as follows:

(3) $$n_{i}=\sum_{k=1}^{|N_{i}|}\left(1-q_{k}\right)$$

where $N_{i}$ denotes the set of interfered nodes, and $q_{k}$ denotes the link quality of the interfered links. Following the signal-to-noise-ratio (SNR) model [32,33], we estimate $q_{k}$ as follows:

(4) $$ber_{k}=Prob\left[\frac{g_{k}}{ns_{k}}\geq \delta_{a}\right]$$

where $g_{k}$ denotes the received gain of signal, and $ns_{k}$ denotes the overall received noise at node *k*, which includes the received signal strength from the edge server to node *k*.

By combining packet length pl and bit error rate $ber_{a}^{t}$, we can calculate the expected packet reception rate for each sample, $q_{k}$, according to [34]:

(5) $$q_{k}={\left({\left(1-ber_{k}\right)}^{8}+8ber_{k}{\left(1-ber_{k}\right)}^{7}\right)}^{pl/4}.$$

By combining the above equations, the edge server is able to obtain the expected gain of packet forwarding. If the gain is larger than a given threshold, the edge server starts forwarding the packet. Otherwise, the packets is dropped and the edge server continues overhearing.

**Figure 3:**
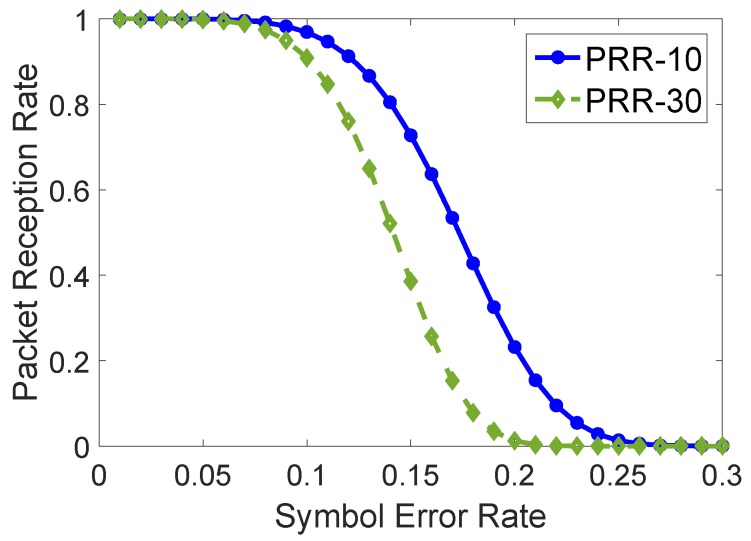
The symbol error rate versus the packet capture probability.

We conduct an experiment to study the relationship between symbol-error-rate (SER) and the packet capture probability (PRR). Figure 3 shows the results. We can infer that the symbol error rate is directly impacted by the environmental noise and the interfering signals from the edge server. Similar results have been observed in [33].

### 3.4. Edge-Aided Retransmissions

When the edge server overhears one packet has been repeatedly retransmitted to the same destination node, it estimates the expected link quality of the target link and then decide whether to retransmit the packet for that link.

We identify the poor links as links with a quality smaller than $t_{p}$. If the edge server overhears the same packet transmission $n_{r}$ times, then it can estimate the link quality of the target link *k* as:

(6) $$q_{k}=\frac{1}{n_{r}+1}.$$

When a poor link is identified, the edge node keeps this link in a set $N_{p}$. When the edge node overhears transmissions to certain destinations, it first estimates the probability that the packet is transmitted on a poor link as follows:

(7) $$p_{k}=\frac{n_{k}^{on}}{n_{k}^{all}}$$

where $n_{k}^{on}$ denotes the number of packet transmissions on the link *k*, and $n_{k}^{all}$ denotes the number of packet transmissions of which the destination node is the same with *k*’s destination node.

### 3.5. Discussion

**Figure 4:**
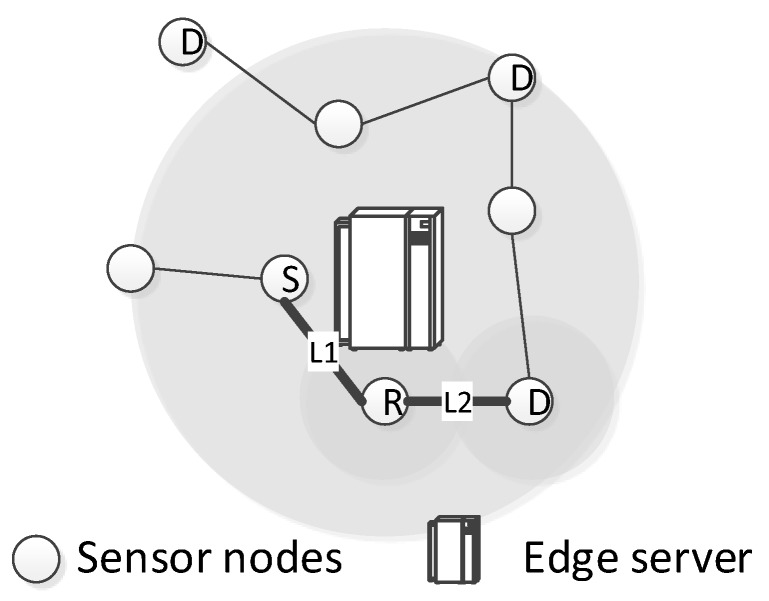
An illustrating example of the impact of asymmetric links in retransmissions.

**Asymmetric links.** One limitation would be that the edge servers cannot sense the packet losses at the receiver side due to the limited communication range of sensor nodes. To deal with this problem, we can employ a multi-hop estimation scheme to estimate the number of retransmissions on an indirect link. Following Equation (6), we can obtain the link quality of any link in the edge server’s communication range. Suppose the target link $l_{1}$ is in the edge’s communication range but the edge is out of its communication range. Another link $l_{2}$ and the edge server are in each other’s communication range and at the same time $l_{1}$ and $l_{2}$ are in each other’s communication range. Our goal is to estimate the link quality of $l_{1}$ via $l_{2}$. Note that $l_{1}$’s sender node is the receiver node in $l_{2}$, as illustrated in Figure 4. $l_{1}$ is the link from node S to R and $l_{2}$ is the link from node R to D. In this case, the feedback of node D cannot be heard by the edge servers.

To notify the edge servers about the link status, we require each sender to add an “additional delay” field in its packet and record the sequence difference for its next hop link. The value of “additional delay” field, $d_{a}$, is calculated as:

(8) $$d_{a}=\tau \left(s_{s}-s_{r}\right)$$

where τ is the duration of a packet transmission. With this information, the edge server will be able to infer how many packets are lost in $l_{1}$ with $d_{a}$ from node S (as S will transmit the difference between $l_{1}$ and $l_{2}$).

**Placement of the edge servers.** The placement of edge servers can affect the performance improvement. Specifically, for the same sensor network, if the edge server is placed in an area covering more sensor nodes, more transmissions can be migrated from sensor nodes to the edge servers. If the edge server is placed in an area covering fewer sensor nodes, there will be less chance to exploit edge servers and thus the improvement is expected to decrease. For deploying an edge server to an existing sensor network, it would be better to place it in a dense area to enhance the chance of communication offload.

**Power consumption.** It is worth noting that the basic idea of our work is to migrate the power consumption from sensor nodes to the edge nodes. The total energy consumption may not be reduced or even be increased. The reason is although a smaller number of packets are transmitted by the sensor network, the transmission power of edge servers is much larger than that of the sensor nodes. However, considering that the sensor networks are powered by batteries, the power migration can effectively enlarge the lifetime of sensor networks.

**Security communications.** To avoid security risks in the sensor-to-sensor and sensor-edge communications, we can apply a series of different cryptography and authentication techniques that are designed for resource-constrained devices [35,36].

## 4. Evaluation

In this section, we conduct simulation experiments to study the performance of the proposed edge-aided data transmissions in sensor networks.

### 4.1. Experimental Settings

**Figure 5:**
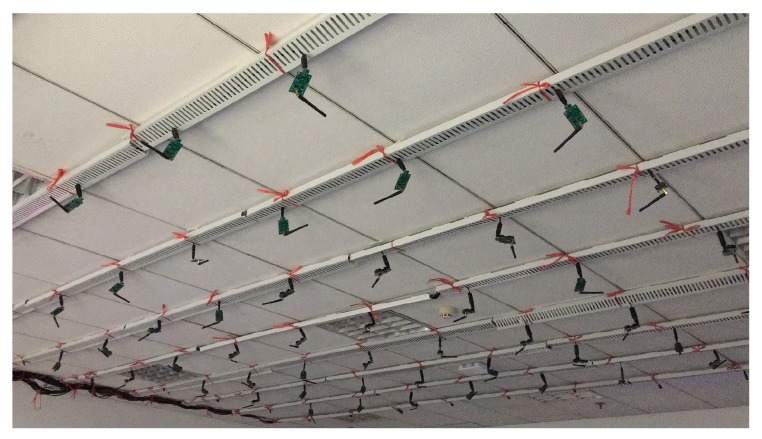
The experimental testbed with 8 × 10 sensor nodes.

We use both testbed and simulation experiments to study the performance of the proposed edge-aided transmission. The testbed (Figure 5) contains 8 × 10 sensor nodes (MSP430+CC2420), and the radio power is set to level one to form an eight-hop network. We assign three to four nodes as edge servers and set the transmission power to level two (the coverage is three times larger). Each node calculates its own transmissions and then sends the statistics to a central server via serial ports. In testbed experiments, we compare our work with the recent proposed work in [4], which considers different link features to find out the best forwarders in the process of data dissemination. We mainly study the key metrics for dissemination—transmission count, which denotes the total number of transmissions used for each packet. The number includes both transmissions from edge server and sensor nodes. Since we have additionally employed edge servers in the data transmission, the transmission count is expected to be reduced. We repeat the experiments 1000 times to obtain a stable representative result for the work.

To further study different conditions with varying edge servers, we use the TOSSIM [37] simulator to generate sensor networks and then deploy different numbers of edge nodes to the sensor network. Each edge node covers ten sensor nodes with three hops on average, i.e., the ten nodes covered by the edge server have three hops. The data transmissions follow the framework proposed in Section 3. We tune the following parameters to comprehensively study the performance of edge-aided transmission. Each simulation with different settings is repeated 1000 times.

- The number of edge servers deployed in the network. When there are more edge servers, there is more chance of forwarding and retransmissions from the edge servers. The overall performance is expected to be improved. When there are fewer edge servers, there is less chance of forwarding and retransmissions from the edge servers, and the overall performance is expected to be degraded;
- The number of destination nodes for each packet. We tune the number of destination nodes for each packet. When there are more destination nodes, more transmissions will be required and there will be more potential for edge-aided optimizations. Otherwise, fewer transmissions will be required and there will be less potential for edge-aided optimizations.

With the above variations, we proceed to study two end-to-end metrics: transmission count and transmission delay.

### 4.2. Evaluation Results

**Figure 6:**
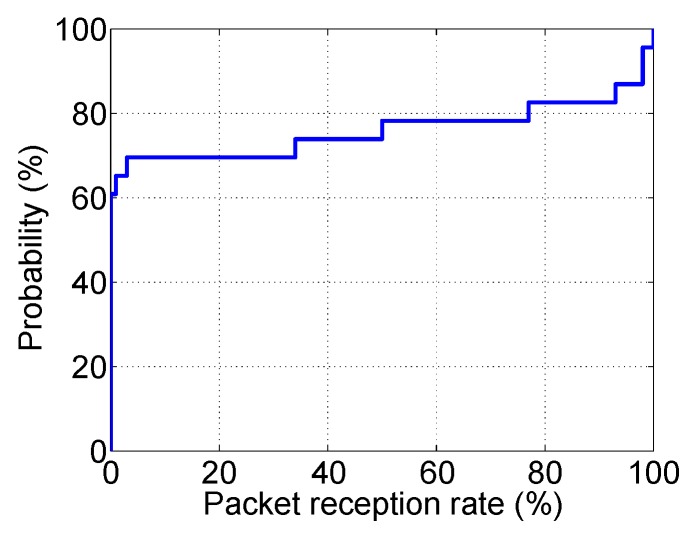
The link quality cumulated distribution function (CDF) of the testbed.

The link quality distribution of the testbed is shown in Figure 6. From the cumulated distribution function (CDF) of link quality values in the network, we can see that 80% of links have link quality smaller than 80%, which means 20 packets are likely to be lost in every 100 transmissions.

**Figure 7:**
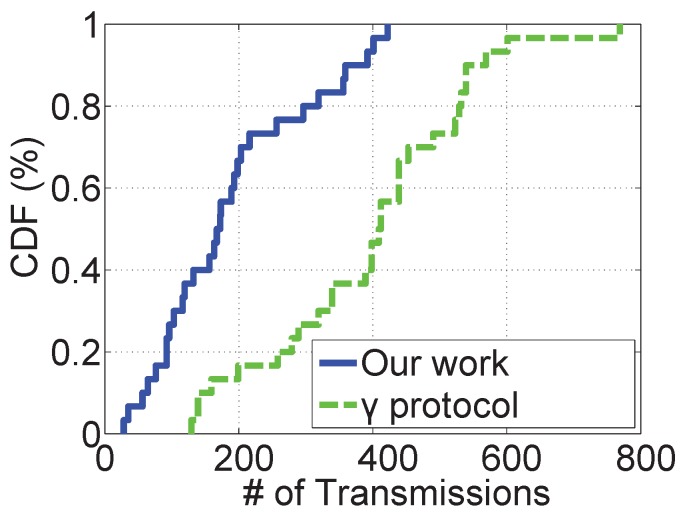
The transmission count compared to the existing work.

We first compare our work with the existing work, γ protocol, proposed in [4]. Figure 7 shows the CDF of transmission counts in both our work and the γ protocol. First, the transmission count of most network nodes is smaller than 400 with our work while only around 60% of nodes have a transmission count smaller than 400, and nearly 30% of nodes have a transmission count larger than 500. We can see that all network nodes have much fewer transmissions compared to the γ protocol. Second, some nodes have more transmissions compared to other nodes. We analyze the experiment logs and identify that the sensor nodes that are not covered by edge servers have more transmissions compared to the nodes covered by edge servers. The reason is that the nodes covered by edge servers have a much larger chance to offload their transmission tasks to the edge servers and thus the transmission count is reduced.

**Figure 8:**
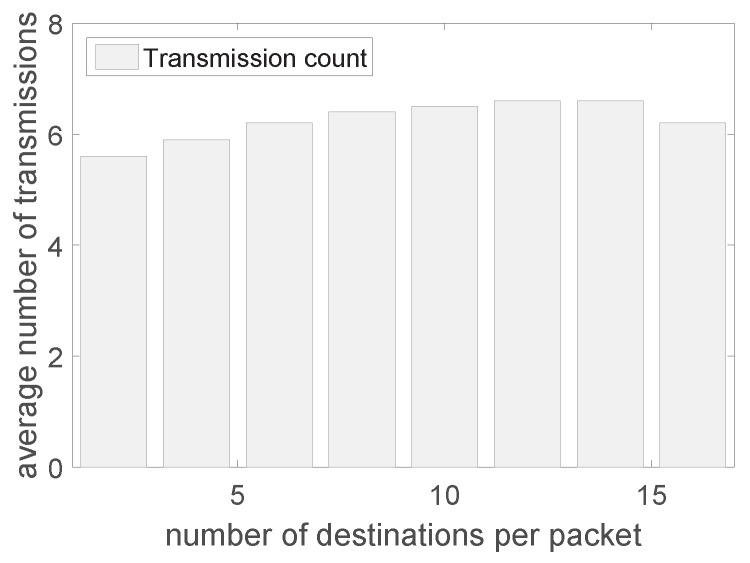
The transmission count with varying number of destination nodes.

**Figure 9:**
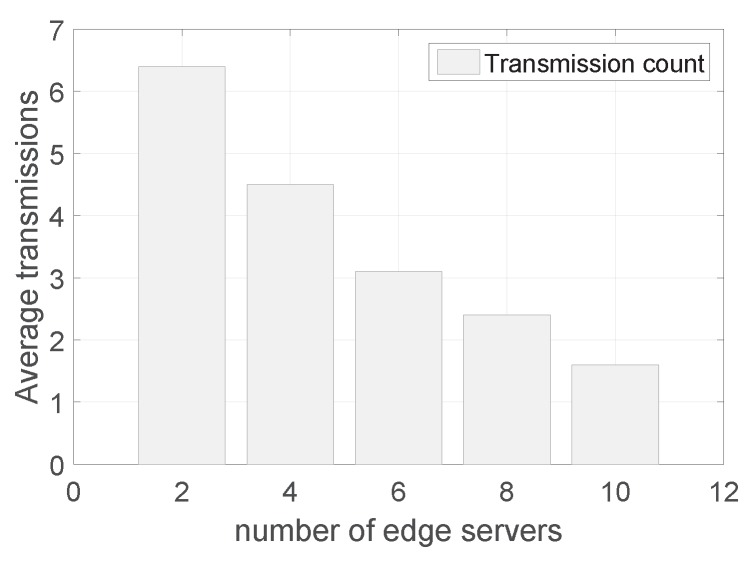
The transmission count with a varying number of edge servers.

Figure 8 shows the transmission count with varying number of destination nodes. We set the number of edge nodes as two in this experiment (we change the number of edge nodes later in Figure 9). We can see that when there are more destinations for each packet, more transmissions are required. It is reasonable because more destinations need more forwarding of the packets on more hops. In addition, the transmissions grow linearly in general. The reason is that we randomly set the destinations for each packet, thus the average hops for each destination is similar. We also notice that when the number of destinations is too large (>14), the number of transmissions decreases because when there are too many destinations, those destinations are close to each other and can be covered by the same transmissions (due to the nature of wireless broadcast).

To clearly understand the contributions of each building block in the proposed framework, we then conduct simulations to separately evaluate the performance of forwarding and retransmissions.

Figure 9 shows the transmission count with a varying number of edge servers. We can see that as the number of edge servers increases, the number of packet transmissions decreases because the edge servers’ forwarding and retransmissions can save a number of transmissions. Additionally, the degradation of the transmission count becomes smaller as the number of edge servers increases. The reason is that when when there are few edge servers, they are deployed without any overlapped areas and each additional edge server is able to cover a large number of sensor nodes. When there are many edge servers, there is overlap among different edge servers and thus the transmission reduction becomes smaller. From the results, one can also tell how many edge servers should be deployed given the level of transmission efficiency in sensor networks.

**Figure 10:**
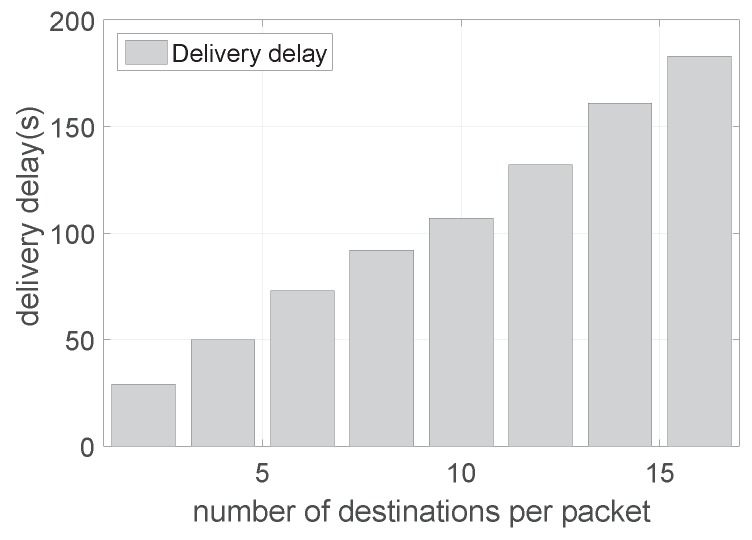
The delivery delay with a varying number of destination nodes.

Figure 10 shows the delivery delay with varying number of destination nodes. The delivery delay is calculated as the time difference from the source node to the destination nodes via multiple hops. We can see that the delay increases almost linearly along with the number of destination nodes. However, different from transmissions, the delay continues increasing when there are more than 14 destinations. The reason is that unlike transmissions, the delivery delay needs to account for the feedback delay from all destinations. Although packet transmissions can arrive at different destinations at the same time, their ACKs cannot be sent back to the sender at the same time. As a result, the delivery delay continues to increase as the number of destinations increases.

**Figure 11:**
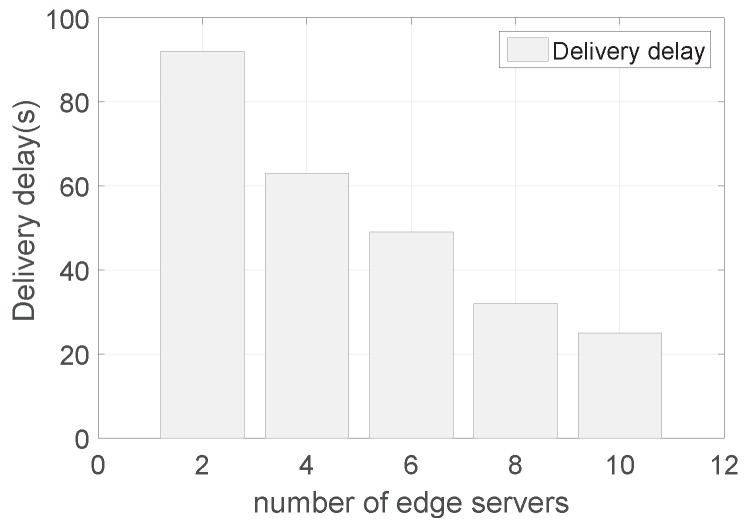
The delivery delay with a varying number of edge nodes.

Figure 11 shows the delivery delay with a varying number of edge nodes. Similar to the transmission count, the delivery delay decreases as the number of edge nodes increases. The reason is that with more edge servers, there is more chance to exploit the edge servers for forwarding and retransmissions. As the communication coverage of edge servers is much larger than that of sensor nodes, the delay on multiple hops is saved. Furthermore, the reduction also becomes smaller as the number of edge servers becomes larger. The reason is that when there are too many edge servers, most of the transmissions are performed by the edge servers and there is less optimization space for the delivery delay of transmissions in sensor networks.

## 5. Conclusions

In this paper, we investigate the problem of data transmissions in edge-aided sensor networks. Unlike the existing works that exploit the computing resources of edge servers, we use the communication resources of edge servers to significantly reduce the transmission and delay overheads of data transmissions in sensor networks. We first adaptively employ edge servers for data forwarding to save transmissions and then identify poor links by multi-hop overhearing. By retransmitting packets for the temporarily poor links, the transmission and delay overheads can be further reduced. We conduct simulation experiments and the results show that the performance of data transmission in sensor networks is largely improved in terms of both transmission count and delivery delay.

## References

[1] Fadel E., Gungor V., Nassef L., Akkari N., Maik M.A., Almasri S., Akyildiz I.F. (2015). A survey on wireless sensor networks for smart grid. Comput. Commun..

[2] Zhao Z., Xu X., Dong W., Bu J. (2015). An Accurate Link Correlation Estimator for Improving Wireless Protocol Performance. Sensors.

[3] Mo L., He Y., Liu Y., Zhao J., Tang S., Li X., Dai G. Canopy Closure Estimates with Greenorbs: Sustainable Sensing in The Forest. Proceedings of the 7th ACM Conference on Embedded Networked Sensor Systems.

[4] Zhao Z., Dong W., Bu J., Gu T., Min G. (2017). Accurate and generic sender selection for bulk data dissemination in low-power wireless networks. IEEE/ACM Trans. Netw..

[5] Chen Y., Shen W., Huo H., Xu Y. A Smart Gateway for Health Care System Using Wireless Sensor Network. Proceedings of the 2010 Fourth International Conference on Sensor Technologies and Applications.

[6] Gao W., Zhao Z., Min G., Cao Y., Duan H., Liu L., Long Y., Yin G. (2017). Link quality aware channel allocation for multichannel body sensor networks. Pervasive Mob. Comput..

[7] Li M., Lin H.J. (2015). Design and Implementation of Smart Home Control Systems Based on Wireless Sensor Networks and Power Line Communications. IEEE Trans. Ind. Electron..

[8] Magno M., Polonelli T., Benini L., Popovici E. (2015). A Low-cost, Highly Scalable Wireless Sensor Network Solution to Achieve Smart LED Light Control for Green Buildings. IEEE Sens..

[9] Mao S., Leng S., Zhao Q., Zhao Z. Joint Power and Time Resource Optimization in Full-Duplex Wireless-Powered Communication Networks. Proceedings of the 2017 IEEE International Conference on Internet of Things (iThings) and IEEE Green Computing and Communications (GreenCom) and IEEE Cyber, Physical and Social Computing (CPSCom) and IEEE Smart Data (SmartData).

[10] Xi W., Zhao J., Li X.Y., Zhao K., Tang S., Liu X., Jiang Z. Electronic frog eye: Counting crowd using wifi. Proceedings of the 2014 IEEE Conference on Computer Communications.

[11] Zhao Z., Zhao Z., Min G., Shu C., Wang Z., Duan H. Non-Intrusive Biometric Identification for Personalized Computing Using Wireless Big Data. Proceedings of the 2018 IEEE SmartWorld, Ubiquitous Intelligence & Computing, Advanced & Trusted Computing, Scalable Computing & Communications, Cloud & Big Data Computing, Internet of People and Smart City Innovation (SmartWorld/SCALCOM/UIC/ATC/CBDCom/IOP/SCI).

[12] Morabito R., Cozzolino V., Ding A.Y., Beijar N., Ott J. (2018). Consolidate IoT edge computing with lightweight virtualization. IEEE Netw..

[13] Sun X., Ansari N. (2016). EdgeIoT: Mobile edge computing for the Internet of Things. IEEE Commun. Mag..

[14] Mao Y., Zhang J., Letaief K.B. (2016). Dynamic computation offloading for mobile-edge computing with energy harvesting devices. IEEE J. Select. Areas Commun..

[15] Wang F., Xu J., Wang X., Cui S. (2018). Joint offloading and computing optimization in wireless powered mobile-edge computing systems. IEEE Trans. Wirel. Commun..

[16] Wang Z., Zhao Z., Min G., Huang X., Ni Q., Wang R. (2018). User mobility aware task assignment for mobile edge computing. Future Gener. Comput. Syst..

[17] Chen M., Hao Y. (2018). Task offloading for mobile edge computing in software defined ultra-dense network. IEEE J. Select. Areas Commun..

[18] Abbas N., Zhang Y., Taherkordi A., Skeie T. (2018). Mobile edge computing: A survey. IEEE Int. Things J..

[19] Zhao Z., Min G., Gao W., Wu Y., Duan H., Ni Q. (2018). Deploying edge computing nodes for large-scale IoT: A diversity aware approach. IEEE Int. Things J..

[20] Ganti R.K., Jayachandran P., Luo H., Abdelzaher T.F. Datalink streaming in wireless sensor networks. Proceedings of the 4th International Conference on Embedded Networked Sensor Systems.

[21] Liang C.J.M., Priyantha N.B., Liu J., Terzis A. Surviving wi-fi interference in low power zigbee networks. Proceedings of the 8th ACM Conference on Embedded Networked Sensor Systems.

[22] Zhao Z., Dong W., Chen G., Min G., Gu T., Bu J. (2017). Embracing corruption burstiness: Fast error recovery for zigbee under wi-fi interference. IEEE Trans. Mob. Comput..

[23] Yick J., Mukherjee B., Ghosal D. (2008). Wireless sensor network survey. Comput. Netw..

[24] Gnawali O., Fonseca R., Jamieson K., Moss D., Levis P. Collection tree protocol. Proceedings of the 7th ACM Conference on Embedded Networked Sensor Systems.

[25] Dong W., Liu Y., Zhao Z., Liu X., Chen C., Bu J. (2014). Link quality aware code dissemination in wireless sensor networks. IEEE Trans. Parallel Distrib. Syst..

[26] Zhao Z., Dong W., Bu J., Gu Y., Chen C. (2015). Link-correlation-aware data dissemination in wireless sensor networks. IEEE Trans. Ind. Electron..

[27] Zhao Z., Dong W., Bu J., Gu T., Chen C., Xu X., Pu S. Exploiting link correlation for core-based dissemination in wireless sensor networks. Proceedings of the 2014 Eleventh Annual IEEE International Conference on Sensing, Communication, and Networking (SECON).

[28] Zhao Z., Bu J., Dong W., Gu T., Xu X. (2016). CoCo+: Exploiting correlated core for energy efficient dissemination in wireless sensor networks. Ad Hoc Netw..

[29] Zhao Z., Wang Z., Min G., Cao Y. (2017). Highly-efficient bulk data transfer for structured dissemination in wireless embedded network systems. J. Syst. Archit..

[30] Cheng L., Niu J., Luo C., Shu L., Kong L., Zhao Z., Gu Y. (2018). Towards minimum-delay and energy-efficient flooding in low-duty-cycle wireless sensor networks. Comput. Netw..

[31] Chen G., Dong W., Zhao Z., Gu T. Towards accurate corruption estimation in zigbee under cross-technology interference. Proceedings of the 2017 IEEE 37th International Conference on Distributed Computing Systems (ICDCS).

[32] Andrews M., Dinitz M. Maximizing capacity in arbitrary wireless networks in the SINR model: Complexity and game theory. Proceedings of the IEEE INFOCOM 2009.

[33] Zhao Z., Dong W., Guan G., Bu J., Gu T., Chen C. Modeling link correlation in low-power wireless networks. Proceedings of the 2015 IEEE Conference on Computer Communications (INFOCOM).

[34] Dubois-Ferrière H., Estrin D., Vetterli M. Packet combining in sensor networks. Proceedings of the 3rd International Conference on Embedded Networked Sensor Systems.

[35] Eisenbarth T., Kumar S., Paar C., Poschmann A., Uhsadel L. (2007). A survey of lightweight-cryptography implementations. IEEE Des. Test Comput..

[36] Farash M.S., Turkanović M., Kumari S., Hölbl M. (2016). An efficient user authentication and key agreement scheme for heterogeneous wireless sensor network tailored for the Internet of Things environment. Ad Hoc Netw..

[37] Levis P., Lee N., Welsh M., Culler D. TOSSIM: Accurate and scalable simulation of entire TinyOS applications. Proceedings of the 1st International Conference on Embedded Networked Sensor.

